# Binocular summation is affected by crowding and tagging

**DOI:** 10.1038/s41598-021-83510-8

**Published:** 2021-03-01

**Authors:** Ziv Siman-Tov, Maria Lev, Uri Polat

**Affiliations:** School of Optometry and Vision Sciences, Bar-Ilan University, Ramat Gan, Israel

**Keywords:** Neuroscience, Visual system, Pattern vision

## Abstract

In perceptual crowding, a letter easily recognized on its own, becomes unrecognizable if it is surrounded by other letters, an effect that confers a limit on the visual processing. Models assume that crowding is a hallmark of the periphery but that it is almost absent in the fovea. However, recently it was shown that crowding occurs in the fovea of people with an abnormal development of functional vision (amblyopia), when the stimulus is presented for a very short time. When targets and flankers are dissimilar, the crowding is reduced (tagging). Since a combination of binocular inputs increases the processing load, we investigated whether color tagging the target reduces crowding in the fovea of subjects with normal vision and determined how crowding is combined with binocular vision. The crowding effect at the fovea was significantly reduced by tagging with a color target. Interestingly, whereas binocular summation for a single letter was expected to be about 40%, it was significantly reduced and almost absent under crowding conditions. Our results are consistent with the notion that the crowding effect produces a high processing load on visual processing, which interferes with other processes such as binocular summation. We assume that the tagging effect in our experiment improved the subject's abilities (sensitivity and RT) by creating a "segmentation", i.e., a visual simulated separation between the target letter and the background. Interestingly, tagging the target with a distinct color can eliminate or reduce the crowding effect and consequently, binocular summation recovers.

## Introduction

In vision, local features are grouped to produce a meaningful percept. This process is context dependent and requires integration between remote image parts obeying the Gestalt theory of perception^1,2^; it is assumed to operate at a very early (pre-attentive) stage of processing, without involving attention. Thus, a contextual effect modifies the appearance of local patterns when they appear between other patterns. Center-surround, grouping, crowding, and pop-out are considered as a context effect^3^.

It is suggested that the first step in the feature integration is a preattentive stage^4,5^, which gathers information about basic features in the scene, followed by a second stage that combines the local features (grouping) of an object, to perceive the whole object; it requires focused attention. If the local and basic features comply with the Gestalt principles, they can be grouped together. However, when some features differ from the others, they stand-out (pop-up) and the grouping process is disrupted. Thus, two main phenomena of visual integration, namely, crowding and pop-out, seem to be antagonistic in the processing. Crowding occurs with grouping; it minimizes local information, emphasizing the whole, whereas pop-out breaks up the grouping and enhances local information. Interestingly, most crowding and pop-out studies are performed at the periphery (see below).

Visual crowding is the inability to recognize objects in clutter and sets a fundamental limit on conscious visual perception and object recognition throughout most of the visual field^3,6^; it is most pronounced in peripheral vision or in the fovea of people with strabismic amblyopia and can block an ordinarily visible stimulus from visual awareness^3,6–9^. According to contemporary models, crowding occurs at multiple stages in the visual hierarchy^10,11^. It begins at an early stage of visual processing; it occurs when the target and flank overlap within the same neural unit^3,8^, or with pooling^12^; top-down effects occur without a clear role of attention^13^. Two relevant theories are the Attentional Resolution theory, suggesting that peripheral crowding can be reduced by cueing^14,15^ and the Configural Grouping theory (Gestalt principles)^16^, which assumes that crowding occurs when a similarity exists between targets and flankers^17,18^.

It was suggested that in foveal vision crowding typically only occurs over very small distances (4–6 arc min)^6^ or does not occur at all^19^. A crowding condition at the fovea is very difficult to create because the resolution in the fovea is very high due to the small size of the perceptive field (PF), which is the psychophysical analog to the classical neuronal receptive field in the visual cortex^20^. The fovea has been proposed as the processing unit of human visual perception^3^. However, some crowding studies have been performed at the fovea^21–25^. Most foveal studies explored crowding using configurations of vernier acuity. In addition, foveal crowding in normally sighted subjects was created by using a target that was presented for a very short time^26^. For 0.4 letter-spacing , foveal crowding was remarkable, significant, and apparent for presentation times from 30 to 240 ms^26^. Thus, crowding exists in the fovea, but it decreases as the presentation time increases. Recently it shown that, like at the fovea^27^, crowding at the periphery increases for short presentation times and decreases for longer durations^28^.

A feature search (also known as a "disjunctive" or "efficient" search) is a visual search, the ability to detect a target of interest against a background of distracting objects that differ from the target by a unique visual feature such as color, shape, orientation, size, or motion^29^. The ‘pop out’ of a distinctive element embedded in a regular pattern is a manifestation of the way context affects visual perception. An example of a feature search task is to ask a participant to identify a target surrounded by distractors while the target’s feature is distinct from the distractors^5^. The efficiency of a feature search with regard to reaction time (RT) and accuracy depends on the pop-out effect, as well as bottom-up and parallel processing. However, the efficiency of a feature search can be affected by the number of distractors present^30^, above some critical distractor number (density); when the distance between them decreases, the subject’s RT improves (if it decreases, it is faster)^30,31^. The pop-out effect is a part of a feature search that characterizes the target's ability to stand out from surrounding distractors owing to its unique feature. Thus, pop-out can break up grouping by enhancing local elements. One can consider crowding with a tagged target by color as a type of pop-out in which the subjects are required to identify a target surrounded by distractors^21,31,32^.

On the periphery, crowding is reduced when targets and flankers are dissimilar in shape, size, orientation, polarity, spatial frequency, depth, color, and motion^9,28,33^. The results suggest that with a tagged target (e.g., color and luminance) there is feature-based interaction or salience-based facilitation when the tagged target becomes more salient than the background in directing attention to the target location^33,34^. Kooi et al. (1994)^28^ examined the effect of similarity and duration on contour interaction. The stimulus consisted of a test T surrounded by four flanking elements. The flanks were differentiated from the target in one of several ways: contrast polarity, shape, depth, color, or eye-of-origin. The subject's task was to report the orientation of the target T. For some observers, the condition of the color difference yielded a very significant improvement in correct recognition. However, color differences have a smaller effect than the other conditions. Another study performed a few experiments in the periphery and explored the "effect of color pop-out on the recognition of letters in crowding conditions"^33^. The results of this study showed that both the colored target and the colored blob decrease the effect of crowding, but that the red letter is more effective than the yellow blob in reducing the crowding effect. This result may indicate that with colored letters, there is a feature-based interaction in addition to salience-based facilitation. Alternatively, the red letters may simply be more salient than the yellow blob and therefore, more effective in directing attention to the target location^33^. An additional study used the orientation discrimination task of the central Gabor patch under crowding conditions when the target and flanking were presented in a color similar to and different from each other^34^. Their findings were unequivocal; when the target and flanking annulus are identical in their chromatic content, crowding increases and decreases for different chromatic properties.

However, note that these studies measured the effect of tagging on crowding at the periphery; thus, a tagged target may help by drawing attention to the target location. In conclusion, when the target and flankers are ‘‘ungrouped” from each other by making them dissimilar in a fundamental property such as color, polarity, or depth so that the target ‘‘pops-out”, crowding is greatly reduced^9,21,28,31–33^.

The effect of tagging on crowding effects in the fovea has been investigated to some extent previously. The pop-out effect can be demonstrated in the fovea of strabismic amblyopes who typically have extensive crowding that largely can be reduced by target tagging (pop-out)^6,35^. This effect could be explained by the notion that the fovea of strabismic amblyopes functionally behaves like the periphery and may have large perceptive fields (the perceptual description of receptive field)^8^ and that tagging the target letter enables a pop-out of the target letter, thus breaking the grouping and reducing the crowding effect in the fovea^8^. Sayim et al. (2008) measured vernier acuity thresholds under foveal crowding conditions, aiming to explore the crowding effect under three different conditions between the target and the flankers: by changing the contrast polarity, color, and by creating stereoscopic depth^21^. They found that the crowding effect was reduced in each of the above experiments. In conclusion, when the target and flankers are ‘‘ungrouped” from each other by making them dissimilar in a fundamental property such as color, polarity, or depth so that the target ‘‘pops-out”, crowding is greatly reduced^33^ at the fovea and periphery.

We noted that there are many similarities between crowding and pop-out. Both are affected by the relationships between the target and the distractors—in a direction opposite to the similarities in shape and color^9,28^; both effects increase with the element’s density^26,30^. Crowding is more difficult when the elements are similar^1,18^, but pop-out is more effective when they are dissimilar. Thus, some conditions that seem to increase crowding enhance the pop-out effect. We also noted that both are mostly explored at the periphery without emphasis on monocular or binocular conditions. Thus, herein we hypothesize that pop-out is more effective under conditions when crowding is stronger even at the fovea.

Many binocular neurons respond best when the retinal images are on a corresponding point in the two retinas’ neural basis for the horopter^36^ (the locus of points in space whose images fall on corresponding retinal points—zero disparity^37^). The horopter can be affected by conditions of phoria, fixation distance, asymmetric convergence, size, and the shape of the image caused by ophthalmic lenses^37^. However, many other binocular neurons respond best when they similarly occupy slightly different positions on the retinas of the two eyes (binocular disparity)^36^. The big question is how does the visual system know which image in the right eye belongs to which image in the left eye in order to make a good match between them? This question is also known as the 'correspondence problem'. The problem intensifies for complex stimuli that contain lots of items such as random-dot stereograms (RDS)^38^.

There are several hypothetical ways for visual systems to solve the correspondence problem; some of them are outlined here: (i) Blurring the image, i.e., using only the low-spatial frequency information; this information serves as an anchor for the visual system and helps it to identify which part of the image corresponds to the other, without trying to match every detail in the complex image, between the eyes^39^. (ii) The uniqueness constraint rule^40,41^, i.e., every feature in the world is represented exactly once in each retinal image. (iii) By the continuity constraint rule^38,40^, i.e., except at the edges of objects; neighbouring points in the world lie at similar distances from the viewer.

Crowding and pop-out studies have largely been examined under binocular conditions, probably after the operation of binocular correspondence. Whereas early work^42^ noted that binocular sensitivity exceeded monocular by about 40% (about √2), later studies found that binocular summation can be above √2^43^. Contrast detection at different spatial frequencies under both monocular and binocular conditions affects the summation ratios across spatial frequencies, sometimes above and sometimes below √2^44^. A meta-analysis of 65 studies demonstrated conclusively that binocular summation is significantly greater than the traditional value of √2^43^.

Binocular summation is maximal when stimuli are presented to corresponding retinal loci in both eyes and when the stimuli have coincident onsets^45–47^; it is greatest at low contrasts for briefly presented stimuli, and it is reduced systematically at higher contrasts. Therefore, monocular and binocular thresholds are approximately equal at higher contrasts (above 15%)^48^. Binocular summation for low-contrast stimuli is reduced with increasing presentation time^48^. The interocular suppression for low contrast and short presentation times is small and increases as the contrast increases^49^. However, in orientation discrimination, the interocular suppression decreases as the contrast increases^50^. According to gain-control theory^51^, the gain that each eye exerts on the other eye is proportion to the strength of the input.

How does binocular foveal crowding change with binocular viewing and age? A study of 56 normally sighted children (7–14 years of age) and 22 adults (21–38 years of age)^52^ measured foveal crowding using Landolt C and bars; the authors found that the resolution acuity was better under the binocular viewing condition than under any of the other three viewing conditions. They also found that the crowding effect is reduced with age in the monocular condition, approaching adult levels by 14 years of age. In binocular viewing the crowding effect does decrease with maturation, but the trend was not significant. A recent study was conducted with 46 subjects aged 3–15 years with normal vision under foveal crowding and binocular conditions^53^. Children were asked to detect the direction of the central E, under crowding conditions. The results show a reduced crowding effect with increasing age.

Another study (2015) examined the grouping effect under monocular and binocular conditions^54^. A dot matrix was presented to the subject, who was asked to evaluate whether the dots are denser in the vertical or in the horizontal axis. The dots’ ratio was either equal (producing an ambiguous grouping, with an equal probability of perceiving rows or columns) or it changed the proximity in each direction (producing a percept of rows or columns). The authors found that with ambiguous stimuli, binocular viewing paradoxically slows down the reaction time. The author’s possible explanation for this phenomenon is that under ambiguous stimulation the neuronal noise within the visual system determines the responses.

The fact that binocular conditions might be not better than monocular conditions was also concluded in our recent research showing that binocular summation under foveal crowding was significantly reduced and was almost absent during a very short presentation time (40 ms)^55^. Further work from our lab showed that two eyes are not always better than one. A recent study (2020) examined the spatial–temporal aspects of nystagmus perception, aiming to investigate the mechanisms underlying the deterioration of their visual performance under monocular and binocular conditions^56^. Subjects were asked to detect Gabor at different frequencies and presentation times. It was found that the binocular summation mechanism was impaired in the majority of the nystagmus subjects. Further research in our lab (2020) explored the perception of binocular vision and the target contrast detection of Gabor patches and two collinear flankers at different orientations (0° (180°), 45°, 90°, and 135°) in cases of both distorted (oblique astigmatism) and non-distorted vision^57^. As a result, no significant binocular summation of collinear facilitation was observed.

Thus, although it is well documented that binocular viewing in contrast threshold, acuity, orientation summation^43^, and reaction time (RT)^42^ is better, recent studies have shown that a binocular combination of monocular responses near the chance level imposes more processing demand and slows down the response speed, as measured by the reaction time (RT). Moreover, the question of whether two eyes are better than one has been challenged in cases of contrast sensitivity, crowding and masking^58,59^. This suggests that crowding may occur before the site of binocular combination; therefore, this poses the question of whether the binocular combination increases the processing demand, resulting in a higher crowding effect. Alternatively, binocular facilitation may lead to less crowding. In addition, if pop-out disrupts the crowding before the binocular combination, no additional processing demand will be imposed on the binocular processing.

The purpose of this study is (1) to investigate whether the pop-out effect can occur in the crowding condition even when the subject's attention is already directed to the fovea and (2) to examine the effect of crowding on the sensitivity and reaction times in the fovea, trying to determine whether this effect affects binocular summation, and finally (3) to determine whether changing the tagging of the target will improve the sensitivity, reaction time, and binocular summation. To reduce the set-up’s complexities, we investigated these effects at the fovea because attention is directed to it and the binocular combination is well studied. We designed a set of experiments to test the effect of tagging on monocular and binocular crowding at different presentation times. The results show that tagging the target with a distinct color can eliminate or reduce the crowding effect and can recover binocular summation.

## Results

### Preface

We ensured that our subjects (N = 10) had equal visual acuity in both eyes (by "setting the threshold" test). This condition was important to understand how the binocular summation system works under crowding conditions independent of the eye’s differences. A recent study showed that visual acuity decreases as the presentation time decreases^60^; thus, we ensured that the visual acuity is at least 20/20 in each eye for all tested subjects and at a very short presentation time (40 ms). We found that there is no significant difference in sensitivity between the eyes for each condition, color, and presentation time (Two-Factor ANOVA: Single E: *p*(black) = 0.899, *p*(red) = 0.721. Crowded: *p*(black) = 0.262, *p*(red) = 0.989), indicating that the performance of both eyes was similar; therefore, "monocular" in this article refers to the monocular average.

### Condition 1: a single letter

The results show (Fig. 2) that no significant difference exists between the sensitivity of monocular for the red and black targets’ letter (*p* = 0.296) (Fig. 2A,B) and RT (*p* = 0.866) (Fig. 2C,D) for all four different presentation times.

### Condition 2: crowded conditions

Figure 2 shows the results under crowding conditions (0.4 letter-spacing); the average difference in sensitivity for the black target decreases significantly for monocular conditions by a d′ of 1.66 (*p* < 0.0001) and even more for binocular, by a d′ of 2.24 (*p* < 0.0001). RT increases significantly by 151 ms for monocular (*p* < 0.0001) and by 163 ms for binocular (*p* < 0.0001). The sensitivity increases (improves) as the presentation time increases, whereas the improvement for the single letter target is faster and larger (for monocular and binocular).

**Figure 1:**
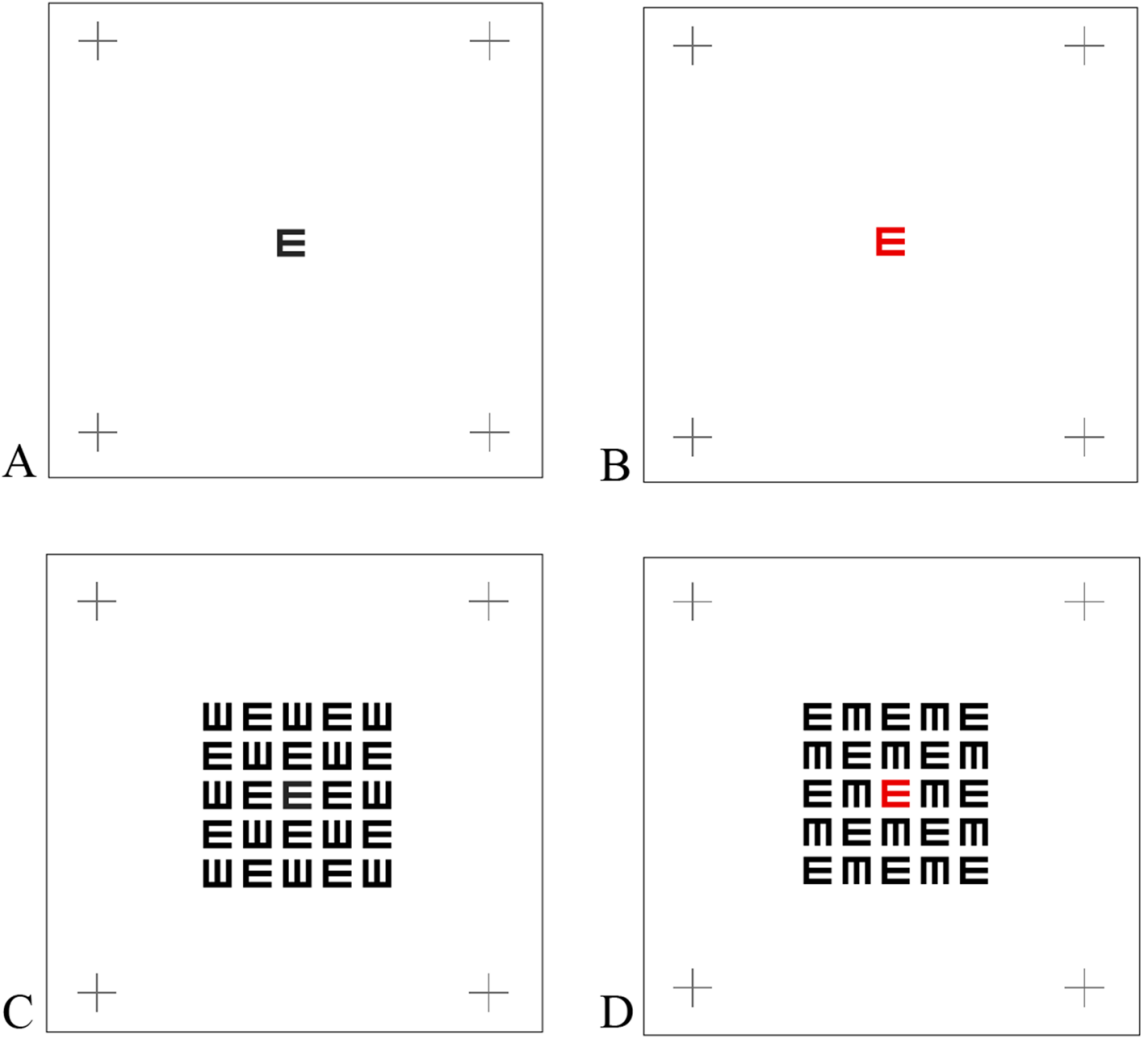
Stimuli: (**A**) A black single letter. (**B**) A red single letter**.** (**C**) A black target letter in a matrix with crowding 0.4 letter spacing. (**D**) A red target letter in a matrix with crowding 0.4 letter spacing.

Additionally, for the red letter crowded condition, the sensitivity also decreased significantly for monocular (*p* < 0.0001) and binocular (*p* = 0.0015), and the RT increased significantly for monocular (*p* = 0.0018) and binocular (*p* = 0.0003); these crowding results are consistent with the study of Lev, Yehezkel and Polat (2015)^26^. However, when comparing the tagged and non-tagged targets in the crowded condition, the crowding effect was reduced or it disappeared by tagging, indicating that the sensitivity improved significantly, compared with the non-tagged condition for monocular (*p* = 0.0003) and binocular (*p* = 0.0001); the RT also improved significantly for monocular (*p* = 0.0018) and binocular (*p* = 0.0095). These effects are found for all four (different) presentation times; however, the improvement with tagging for 40 ms is minor for binocular (*p* = 0.011) and is insignificant for monocular (*p* = 0.295). Thus, the results show that by changing the color of the target letter relative to the background, there is a gain in both accuracy and RT.

### Crowding effect

The crowding effect measures the extent of the sensitivity (d′) and the RT change under the crowded condition, calculated as the difference between the d′ and RT of a single letter target (uncrowded, the zero dashed red line in Fig. 3) to the d′ and the RT of the target under the crowded condition. The crowding effect (d′ and RT) is significantly larger for the non-tagged target (d′; *p* < 0.0001, RT; *p* < 0.0001 monocular, *p* = 0.0001 binocular) than for the tagged target (d′; *p* < 0.0001 monocular, *p* = 0.0015 binocular, RT; *p* = 0.0001 monocular, *p* = 0.0003 binocular). Comparing the monocular-to-binocular conditions of the crowding effect for the tagged target shows that d′ is significantly larger in the binocular than in the monocular (*p* = 0.015); however, the RTs are insignificant (*p* = 0.223). Interestingly, for a non-tagged target, there is more of a crowding effect, i.e., a sensitivity reduction of about 2.23 d′ for binocular and 1.65 d′ for monocular (average differences); the RT increased about 163 ms for binocular and 150 ms for monocular (average differences) (Fig. 3A,C); it probably saturates around 240 ms (for d′ (. In contrast, for a tagged target, the crowding effect on binocular and monocular conditions is not significantly different (d′, *p* = 0.317; RT, *p* = 0.365). In the tagging condition, the crowding effect decreased with increasing presentation time, showing an improvement (increase) of d′ and RT (decrease) (Fig. 3B,D). Note that the sensitivity decreases more under binocular conditions.

**Figure 2:**
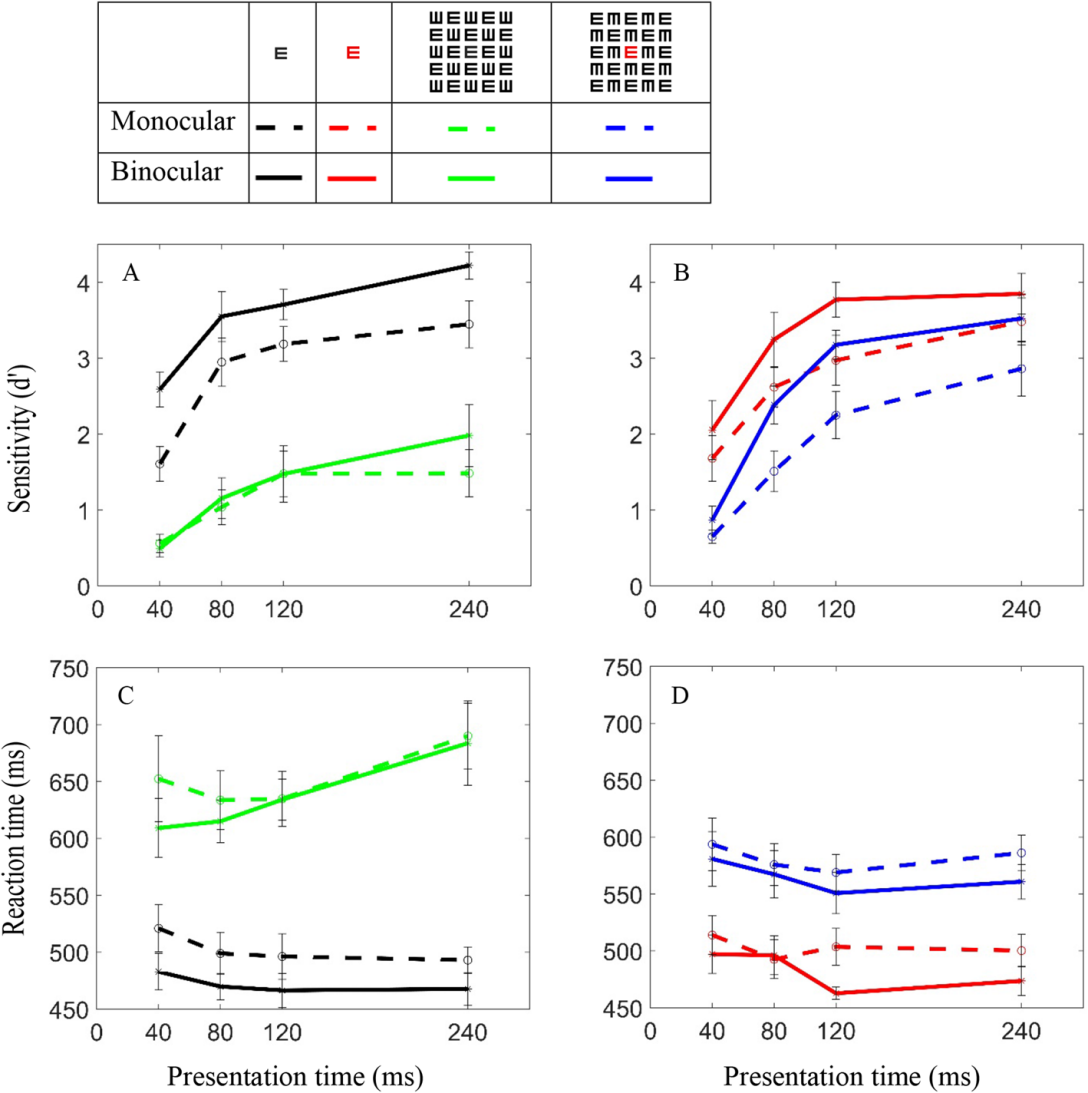
Single letter versus Crowding: Sensitivity: (**A**) for a black target letter. (**B**) For a red target letter. Reaction time: (**C**) for a black target letter. (**D**) For a red target letter. Continuous lines and plus symbols denote binocular vision; dashed lines and circles denote monocular vision. Black denotes a single black target letter and red denotes a single red letter. Green denotes black crowding and blue denotes red crowding. Error bars represent ± SE (standard error) of the mean.

**Figure 3:**
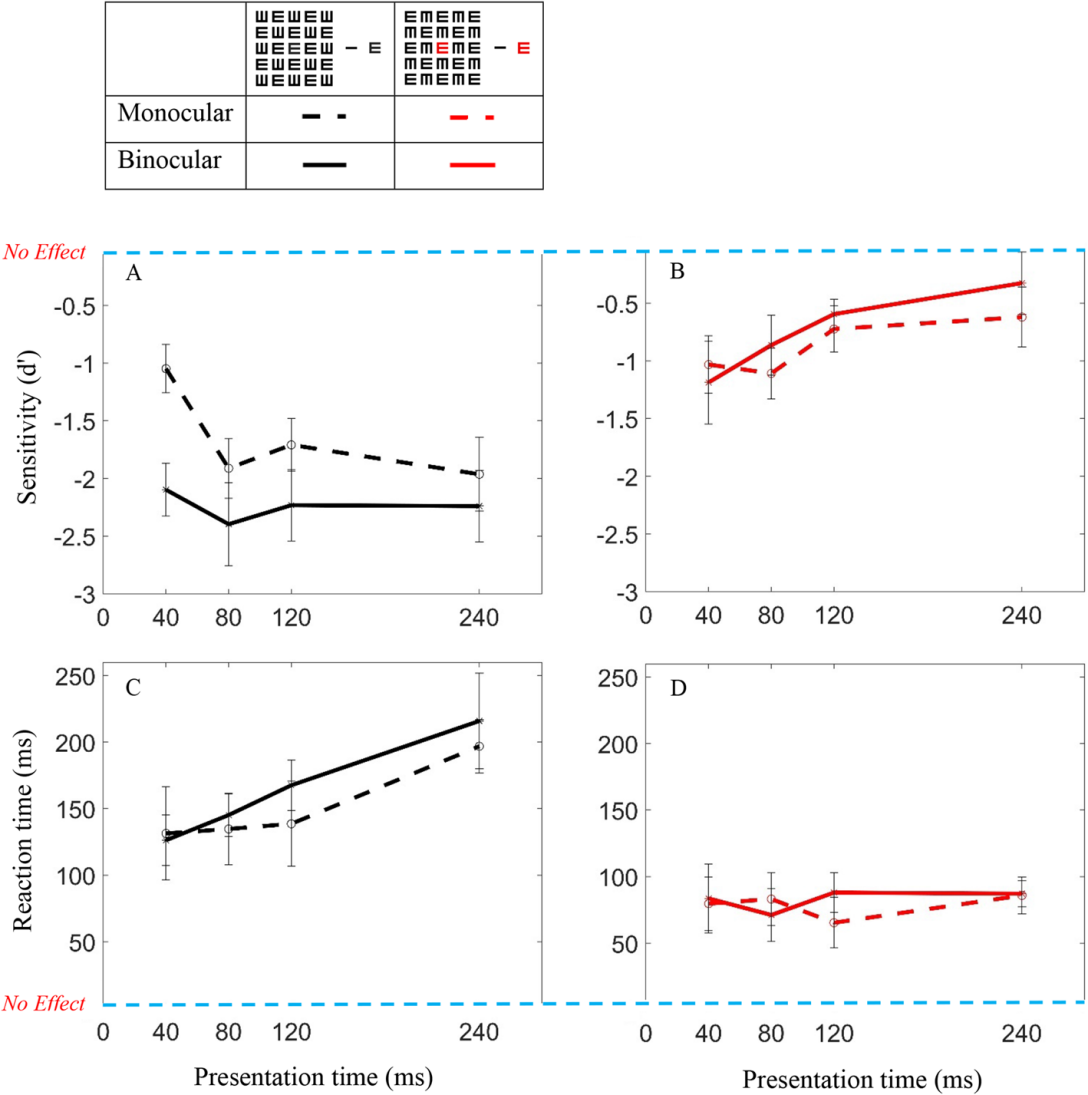
Crowding effect: Sensitivity: (**A**) for a black target. (**B**) For a red target. Reaction time (RT): (**C**) for a black target. (**D**) For a red target. Continuous lines and filled symbols represent binocular vision and dashed lines and open symbols represent monocular vision. Black denotes a black crowding effect and red a red crowding effect. Error bars represent ± SE of the mean.

### Binocular summation

Contrast thresholds are used to calculate the binocular summation, when a binocular-to-monocular ratio of about ~ 1.4 represents a binocular enhancement^42^. A recent meta-analysis study has shown that in some cases, binocular summation is significantly greater than the traditional value of √2 (1.4)^43^. Most of these studies used a target with a near or above contrast threshold; thus, they explored binocular summation in terms of contrast sensitivity. Therefore, in our study we tested the effect of crowding on the binocular summation by calculating the sensitivity (d′) from the hit and false alarm parameters. We calculated the binocular summation as the ratio between the binocular d′ and the monocular d′ (the average of two eyes) (sensitivity ratio; d′_bino_/d′_mono_), similar to previous studies that calculated the binocular summation as the ratio between the monocular to binocular contrast sensitivity^43,56,61^. We believe that this approach, rather than being based on the ratio of the percent correct, enables more direct comparisons with the previous studies.

Table 1 presents the binocular summation ratio at all presentation times under all conditions: The binocular ratio for the black single letter condition was between 1.16 and 1.61; for a red single letter it was between 1.11 and 1.27. Thus, it’s within the range of binocular summation ratio that is showing in the previous studies^42,43^. However, interestingly, for the black crowded conditions the binocular ratio was between 0.88 and 1.34, whereas it approached 1 for 40, 80, and 120 ms, suggesting no summation. Note that for the long presentation time (240 ms), the binocular summation has recovered. Thus, the difference between the monocular and binocular summation was not significantly different (*p* = 0.415). For the red letter it was between 1.23 and 1.58, showing that the binocular summation recovered, and that the difference between monocular and binocular summation is significant (*p* < 0.0001).

**Table 1:** Binocular summation ratio for all presentation time under all conditions.

Presentation time (ms)	Black Single E	Red Single E	Black Crowding	Red Crowding
40	1.61	1.22	0.88	1.34
80	1.20	1.24	1.11	1.58
120	1.16	1.27	1.00	1.41
240	1.22	1.11	1.34	1.23

## Discussion

The purpose of this study was (1) to investigate whether the pop-out effect can occur in the crowding condition even when the subject's attention is already directed to the fovea and (2) to examine the effect of crowding on the sensitivity and reaction times in the fovea, trying to determine whether this effect affects binocular summation, and finally (3) to determine whether changing the tagging of the target will improve the sensitivity, reaction time, and binocular summation. The results show that a combination of monocular inputs in some difficult tasks increases the load on binocular processing. The remarkable monocular crowding at the fovea was not reduced under binocular conditions, suggesting that no binocular summation took place. Interestingly, pop-out reduced the crowding significantly under monocular and binocular conditions.

### Foveal crowding

It was suggested that for very short presentation times at the fovea, larger perceptive fields are activated first, despite the fact that the fovea has very small perceptive fields^3,26^. Thus, our results can be viewed as a simulated condition of peripheral vision at the fovea, which reveals the crowding effects. We found that crowding remarkably and significantly reduces the sensitivity at all presentation times, under monocular and binocular conditions )*p* < 0.0001). The sensitivity under crowding conditions increases as the presentation time increases (it probably saturates at around 240 ms); this supports the hypothesis that whereas inhibition is rapid and transient, following the onset and offset of the stimulus more precisely, the excitation develops slowly and is sustained, lagging behind the stimulus both at the onset and offset^62^. We also found a significant difference (*p* > 0.0001) in RT for all presentation times; this means that the RTs are longer (slower) under crowding conditions. These effects of foveal crowding may be explained by activation of larger perceptive fields at a short presentation time^3^.

### Tagging or pop-out

Many studies have explored the effect of pop-out on crowding at the periphery^28,32–34^, assuming that attention is not allocated to the periphery. Because attention is naturally allocated to the fovea, not many studies of crowding and pop-out were performed at the fovea^21,24,25^. Bonneh et al. (2003) performed an experiment at the fovea on subjects with amblyopic eyes. They found that color tagging the target reduced crowding. However, it is assumed that the amblyopic fovea was functionally behaving like the periphery^8^. Our results show that both the crowding and pop-out effects can be revealed at the fovea when using short presentation times. We tagged the target letter with red under crowding conditions. There was a significant improvement in the sensitivity at all presentation times; it increased as the presentation time increased (*p*(monocular) = 0.0003, *p*(binocular) = 0.0001).

For 40 ms, a minor improvement was found. A possible explanation for this can be found in grapheme-color synesthesia, one of the most common forms of synesthesia (a relatively rare condition in which sensory stimuli cause unusual additional experiences) in which viewing letters or numbers elicits the experience of colors^63^. However, Hubbard and Ramachandran (2005) found that this phenomenon does not occur for short presentation times (at 28 and 56 ms). It was shown that the lateral suppression is dominant in short presentation times^3,62,64^ and may explain the the extent of the interaction (EoI) size (the critical spacing between targets and flankers needed to create a crowding condition)^65^.

A recent study examined whether crowding was reduced on the periphery, when targets and flankers are dissimilar in polarity, under different duration times^66^. It was found that when the stimuli are briefer than these critical durations (an average of 54 ms), the resulting EoIs remain roughly constant. Additionally, the EoIs are smaller, on average, by about 30% when the target and flankers have opposite contrasts; however, a smaller improvement (29%) was observed for the shorter display times (shorter than about 70 ms) that increased (until 44%) as the display time increased (until 213 ms). Thus, it is possible that the color saliency decreases for short presentation times. Tripathy et al. study (2014) confirms the hypothesis that the critical time needed to overcome crowding, at the periphery^66^, depends on the dynamics of excitation (E) and inhibition (I) and on the time that it takes to reach an E/I balance at an optimal suppression level^3^. This result is consistent with our result and a previous study^26^.

The fact that color tagging reduces the crowding effect and raises the sensitivity to close to a single-letter level strengthens the hypothesis that crowding is affected by the properties of target-flankers’ similarity (the Grouping effect)^16–18,21^; therefore, color tagging breaks up the grouping and reduces the crowding effect. Our results are consistent with those of Kooi et al. (1994), who found that peripheral crowding is reduced when targets and flankers are dissimilar in contrast polarity, shape, depth, color, or eye-of-origin^28^; and are also consistent with the results of Westheimer and Herzog (2008), who found a reduction in crowding under foveal color differences and other conditions^21^. We also found results similar to those in the study by Bonneh et al. (2003), performed with amblyopic subjects even though our subjects had normal vision.

Although the pop-out's attention level is generally tested at the periphery, in our experiments the task requires the subjects to direct their attention constantly at the fixation point (target), suggesting the involvement of high brain regions. However, Poletti et al. (2017) showed that selective enhancement of visual processing at the fovea can be restricted to narrow regions and shifted across locations separated by only a few minutes of arc at the fovea^67^. This means that the existing level of attention can be improved to a higher level. These facts can support the hypothesis that crowding occurs at a lower brain area. Although the tagging resembles pop-out, it may have a different mechanism because it takes place in the fovea.

### Reaction time

**Figure 4:**
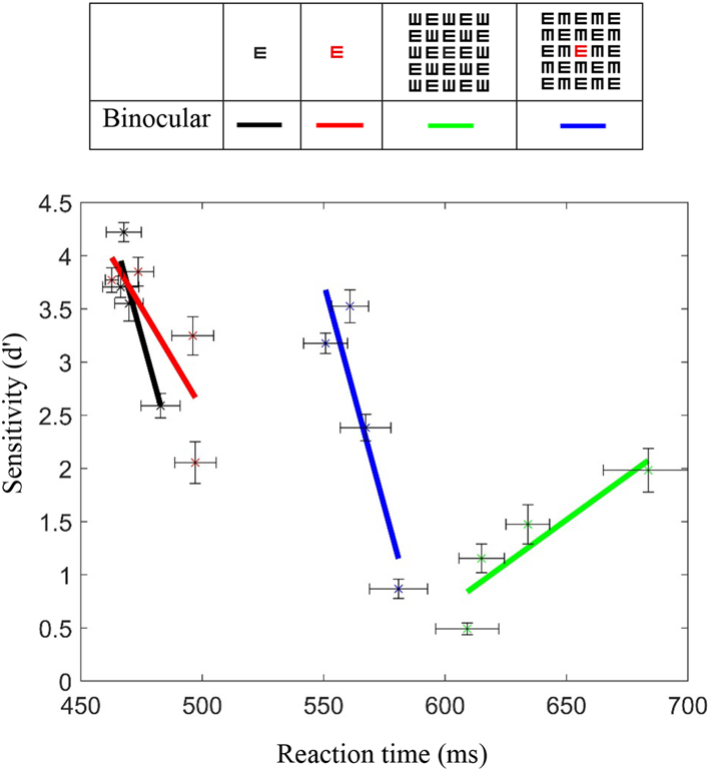
Perceptual load limited capacity: Sensitivity under binocular conditions. The sensitivity trade-off for RT; the processing of letter recognition under crowded conditions (the black letter) requires more processing efforts (the green continuous line). RT SE is represented by the horizontal error bar. d′s SE is represented by the vertical error bar. Error bars represent ± SE of the mean.

As shown in Fig. 2C,D, the RT was longer (slower) for crowded tasks with a black target and shorter (faster) for a red target. In addition, usually a correlation exists between the RT and sensitivity: a short RT for high sensitivity and a long RT for low sensitivity. However, this effect is not true for a crowded condition with a black target (Fig. 4); it was shown that RT did not improve with an improvement in sensitivity (a green solid line). These results clearly show that the processing of letter recognition under crowded conditions (a black letter) requires more processing efforts, as revealed by the longer time needed for decision (RT), i.e., there is a trade-off of sensitivity for RT; this result is in agreement with the empirical speed-accuracy trade-off function^68^.

It was suggested that the visual processing of a briefly presented stimulation, which may induce strong inhibition, increases the processing efforts to rebalance the neural activity (Lev et al. 2015). High perceptual load results in longer RT and higher error rates^69^. Thus, a tagged target may eliminate the strong inhibition, as indicated by the improved RTs. These two facts support the hypothesis that the crowding effect has implications on cognitive functions by posing a bottleneck on the processing of objects and consciousness^9^.

### Binocular vision and the correspondence problem

An additional aim of the study was to determine whether the binocular system provides better and faster detection capabilities (binocular summation) even under crowding conditions.

There may be an issue with binocular correspondence that should be addressed when dealing with binocular summation, i.e., if binocular correspondence should be solved before the binocular summation occurs. Solving the correspondence problem is more straightforward when the target is red and unique, compared to when the target and flankers are black. The correspondence problem is easily solved when there only a single E in the display. However, the correspondence problem might be more difficult to solve when there is an array of 25 elements that are all black. We ensured that the subjects fixate only to the horopter area by checking that everyone has no deviation phoria, asymmetric convergence, and that those subjects with an optical correction had no difference of more than two diopters between the eyes. The stimulus presented on the central fovea and were the location marked by a fixation point. We also maintained a fixed seating distance so that the fixation distance would not change. We performed 'binocular control' (see “Apparatus” in the “Methods”) to ensure that for binocular conditions both eyes focused on the stimulus without deviation or suppression. This includes peripheral lock and binocular fixation at the center. The participants were instructed to start the trial only when the fixation is fused. Moreover, the stimulus has clear boundaries, easy to fuse, and all letters are at zero disparity.

Binocular summation exists when the monocular processing is near the threshold, probably to strengthen weak inputs^43^. However, the efficiency of the binocular summation is impaired for supra-threshold stimuli when the processing approaches the saturation level. Despite the model's prediction that binocular summation is greatest at low contrasts and is reduced systematically with increasing contrast^48^, we found that binocular summation occurs for a single letter at the recognition threshold but at a high contrast (78.4%). This supports the idea that the threshold is determined by the level of neuronal activity when it is weak, regardless of the physical contrast^70,71^. In our experiment, we chose short exposure times and a letter size of the stimulus that induces the performance of a single letter to be visible but near the threshold level, thus enabling binocular summation. Therefore, our single letter experiment results are comparable to those of previous data and models, showing that binocular sensitivity exceeds monocular sensitivity by about 40%^48^.

**Figure 5:**
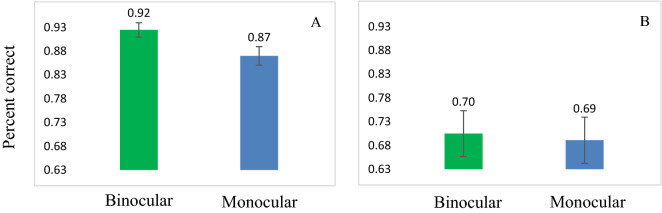
Single letter versus crowding (for a duration time of 120 ms): Percent correct: (**A**) for a single black target letter. (**B**) for black crowding.

Interestingly, we found that binocular summation was impaired in crowding under black conditions. It is important to remember that we determined that the sensitivity of a single letter size for each subject that was above the threshold that was also used for the crowding experiment. Since the crowding condition was more difficult, it reduced the monocular sensitivity to a lower level than that of a single letter. Nevertheless, and surprisingly, binocular summation did not occur for a black single letter. One can claim that at very fast times (40 and 80 ms), no summation is obtained due to the 'floor effect', i.e., the task is very difficult to perform and therefore the subject’s performance collapses to a chance level^72^. This might support the correspondence problem, i.e., when each eye sees a black matrix of an E image, the visual system has difficulty matching the details and indicates the direction of the target letter. However, for red crowding conditions (pop-out), the binocular summation recovered and improved the sensitivity and RT similarly to that of the single letter experiment. However, we believe that this is unlikely because at a longer duration time of 120 ms, the subject’s performance was above chance level (about 1.53 d, 70% percent correct) in both monocular and binocular conditions, yet no binocular summation was observed for the crowded condition (see Fig. 5). Only at longer presentation times was binocular summation of crowding evident (see Fig. 2).

This need for long presentation times for binocular summation for crowded conditions to be observed can probably be explained by several options: (i) The hypothesis for a solution to the correspondence problem, i.e., the visual system blurring the image (the black matrix of E), leaving only the different color information to help (the red target letter); this information serves as an anchor for the visual system and helps it to identify which part of the image corresponds to the other; thus, it helps indicate the direction of the target letter without trying to match every E in the black matrix between the eyes. (ii) As mentioned in the Introduction, sometimes the processing time (RT) is longer in some cases of binocular combination^54^. This is also evident in crowding^26^, both monocular and binocular, and is shown in our data. It is suggested that crowding produces a bottleneck on the processing^6,73^; thus, crowding poses a processing load that may increase in binocular combination. (iii) As suggested before^3,26^, suppression is fast and acts at the first stage of the presentation; thus, it is dominant in short presentation times and is responsible for the crowding effect; facilitation is slower and acts later to release the suppression with increasing presentation time; thus, crowding is not apparent at longer presentation times. (iv) Another possible mechanism^3,26^ is that larger receptive fields are activated first; thus, they process the target and flankers together, and smaller receptive fields start to be involved later (from a coarse to a fine model), thus increasing resolution and enabling the segregation of the target. The fact that binocular summation is apparent only after 120 ms may suggest that these processing times are also applicable for the binocular combination. (v) It is also possible that attention selection takes place at the longer presentation time, to individualize the target location. This possibility seems less likely because the task is foveal and attention is already directed to the clear fixation target.

### Summary and conclusions

First and foremost, our research reinforces the hypothesis that crowding can also occur at the fovea, because we found a significant reduction in sensitivity and a slowdown in reaction times relative to a single letter. The foveal crowding decreases as the presentation time increases. This supports our hypothesis that crowding is affected by the excitation/inhibition balance; inhibition is rapid and transient; it follows the onset and offset of the stimulus more precisely; however, the excitation develops slowly and is sustained, lagging behind the stimulus both at the onset and offset. Thus, inhibition is more dominant at short presentation times, thus exposing the crowding effect^62^.

It was interesting to find that the binocular summation, which improved both the sensitivity and reaction time for a single letter (Fig. 2, black and red lines), was significantly impaired under crowding conditions (Fig. 2, green lines). However, when the target letter was tagged in red, the sensitivity increased and the reaction times were shortened significantly (Fig. 2, blue lines), almost to a single letter level. These results are consistent with the view that crowding is affected by the properties of target-flanker similarity (the Grouping effect); therefore, color tagging breaks up the grouping and reduces the crowding effect^21,28,74^. Although the tagging resembles pop-out, it may have a different mechanism because it takes place in the fovea. Another explanation to consider regarding the absence of the expected binocular summation is the correspondence problem. This problem intensifies for complex stimuli that contain lots of items as does the stimulus in our experiment (matrix of 5 × 5 letters(. It can be assumed that when the target signal is red, the correspondence problem is reduced and the binocular summation recovers. Table 1 may suggest that when the duration is brief (40–120 ms), the correspondence problem is not fully solved and the benefits of summation are not seen yet. When the duration is longer (240 ms) the correspondence problem can be solved showing binocular summation. These findings may contribute to our understanding about the processing of binocular fusion.

Our study cannot rule this out if the absence of binocular summation found here points to deviation from the classical and contemporary binocular summation models or to the correspondence problem. Future studies may address this issue, perhaps by creating a less complex matrix that will maintain the crowding effect; yet, it may significantly reduce the correspondence problem; thus, it may provide better testing of the problem of classical binocular summation versus the correspondence problem.

It was very interesting to find that the crowding effect under binocular conditions was stronger in sensitivity compared with the monocular conditions (Fig. 3A). The above arguments are aimed to discuss the reasons for the absence of binocular summation in crowding; they seem to support the hypothesis that crowding occurs at the monocular level or before the site of binocular combination and poses a bottleneck for binocular processing. This also supports the hypothesis that crowding may occur beyond the site of pop-out, at or before the site of binocular combination.

As expected, our finding highlights the phenomenon of an inverse relationship between sensitivity and response times, i.e., as sensitivity increases, response time decreases. However, this finding is only valid for a single letter (in both colors) and for crowding conditions with target letter tagging. For regular crowding conditions, an opposite linear ratio was obtained, which means that for increased sensitivity, the reaction time also increases. These results clearly show that the processing of letter recognition under crowded conditions (a black letter) requires more processing efforts, as revealed by the longer time needed for a decision, i.e., there is a sensitivity trade-off with the reaction time; this result is in agreement with the empirical speed-accuracy trade-off function^68^.

## Methods

### Participants

Ten adults with normal or corrected-to-normal vision and with no known neurological disorders participated in the study. All subjects had no deviation phoria, asymmetric convergence, and those with optical correction had no difference of more than two diopters between the eyes. The age of the subjects was between 21 and 36 (27 ± 4.53; mean ± STD). Visual functions were estimated by a certified optometrist prior to participation in the study. The participants signed a consent form that was approved by the Internal Review Board (IRB) of Bar-Ilan University and all methods were performed in accordance with the relevant guidelines and regulations and each subject was included only after 'informed' consent was obtained. All the study protocols were approved by the ethics committee of Bar-Ilan University.

### Stimuli and procedures

The sitting distance was fixed at185 cm. We measured the luminance intensity of the stimulus (E letter-single/crowding) and the background (a white screen) using a luminance meter LS-100 (KONICA MINOLTA). The luminance intensity of the target letter was 12.5 cd/m^2^ and consisted of 75 cd/m^2^ of a white screen. The contrasts were 78.4% (calculated for static stimulation); [$\frac{\mathrm{I}-\mathrm{Ib}}{\mathrm{Ib}}$, *I*, *Ib* represent the luminance of the target letter and the background, respectively]. The target letter was an E letter presented at the center of the screen (presented on the central fovea) and was marked by a fixation point. For each subject, the size of the E letter was fitted individually to reach a threshold level of correctly identifying 70–80% correct (a sensitivity of ~ 2 D-prime). The task of the subjects was to indicate the direction of the E target by clicking on the mouse key, right or left. The target letter was shown in black or red. There were 4 conditions: two for a single letter (black, red) and two for crowded conditions (black, red; in the middle of a black matrix) (see Fig. 1). For each condition, the stimulus was presented at four different presentation times (40, 80, 120, and 240 ms) in random order (mixed by trials). All experiments were administered in a dark room and were performed on the same day. Each block lasts about 3–7 min, continuously without a break, but subjects were allowed to take a break without any time limit between the blocks.

#### Setting the threshold

The stimuli were presented for 40 ms (ms), which is the minimum presentation time during all experiments. To choose the right letter size for each subject, a black target (letter E) was displayed in two sizes: 2.7 and 4 (mm), corresponding to a visual acuity of 20/20, 20/30. We chose these sizes to bring the subject to a detection threshold of about 70%. Two experiments (explained below) were conducted while the letter size was constant and did not change. The letter size selected for all subjects and experiments was 2.7 mm.

#### Condition 1: a single letter

The stimuli consisted of a single E, either black or red; the subject’s task was to indicate the direction of the target’s letter by clicking on the mouse key (Fig. 1). A single black E was presented for each presentation time (40, 80, 120, and 240 ms) and eye randomly, mixed by trials, tested on 4 runs for each subject. Thus, there are 60 trials/condition (15 trials for each eye and 15 trials for both in each run × 4 runs = 60 trials/condition for one subject and 600 trials/condition for ten subjects). The same procedure was performed for the single red E. In Fig. 2 each data point is represented by 600 trials and each line by 2,400 trials (4 conditions X 600).

#### Condition 2: crowded conditions

For crowded conditions, in addition to all of the above, there was a matrix of E letters around the target letter, which was arranged randomly. The size of each matrix letter corresponded to the size of the target letter. The size of the matrix was 5 × 5 letters with 0.4 letter spacing between letters. Like in condition 1, the subject's task was to indicate the direction of the target’s letter by clicking on the mouse key. The procedures in the crowded conditions were the same as for the single letter, i.e., there are 60 trials/condition (15 trials for each eye and 15 trials for both in each run × 4 runs = 60 trials/condition for one subject and 600 trials/condition for ten subjects). The same procedure was performed for the red E. In Fig. 2 each data point is represented by 600 trials and each line by 2,400 trials (4 conditions X 600).

### Apparatus

Stimuli were displayed on a 23.5" (53.3 × 30 cm) LCD monitor (ASUS VG248QE) with 1920 × 1080 pixel resolution and at a 120 Hz refresh rate using a NVIDIA GeForce GT 730 graphic card. The visual angle of the LCD monitor was 16.4º X 9.3º. The monitor was designed for gaming and was found suitable for visual psychophysics due to its high temporal accuracy. The stimuli were presented using an in-house-developed platform for psychophysical experiments (PSY) developed by Yoram Bonneh, running on a Windows PC.

We used 3D-Vision-2 Wireless Glasses to control the monocular and binocular vision. The consumer version of NVIDIA 3D Vision consists of wireless LCD shuttered glasses that receive an infrared signal from an emitter connected to a PC via a USB cable. The glasses are shuttered at 120 Hz frequency, updating each eye 60 times per second (60 Hz) for a flicker-free stereoscopic experience^75^. An active shutter 3D system utilizes the technique of displaying stereoscopic 3D images. It works by presenting only an image intended for the left eye, while the right eye views a blank screen; then it presents the right eye’s image while the left eye views a blank screen. This is repeated rapidly so that the interruptions do not interfere with the perceived fusion of the two images into a single binocular image. In this way, the subjects are unaware of the eye whose image is presented. The background luminance was 50 cd/m^2^ with glasses. The direction of the target’s letter and the eye that saw the stimulus (right, left, and binocular) was displayed randomly, and mixed by trials.

#### Binocular control

Four crosses around the stimulus were displayed to the subject during the experiment. Each eye was presented with two crosses (up and down); the experiment was designed so that the subject would see 4 crosses in binocular vision. In normal binocular vision, the subject should always see four crosses during the experiment. The subject was asked to indicate whether the two crosses disappeared or looked double; in these cases, it was decided that the subject could not continue the experiment due to binocular dysfunction.

#### Data analysis

The results of the experiment were presented using the sensitivity index (D-prime, d′). It provides the separation between the means of the signal and the noise distributions (the wrong answers), compared against the standard deviation of the signal or noise distribution. The calculation consists of four parameters: Hit (when the direction of the signal was right and the subject answered correctly), Miss (when the direction of the signal was right and the subject answered incorrectly), Correct Reject (when the signal direction was to the left and the subject answered correctly), and False alarm (when the signal direction was to the left and the subject answered incorrectly).

For each condition we used a Two-Factor ANOVA with repeated measures on both factors. Since the two experiments are different, with different subjects, the comparison was within the same experiments. The above set-up is used for comparisons between conditions.
